# Resistant PRL-secreting PitNET associated with breast carcinoma: a case report and literature review

**DOI:** 10.1007/s13691-024-00741-y

**Published:** 2025-01-04

**Authors:** Roxana-Ioana Dumitriu-Stan, Iulia-Florentina Burcea, Valeria Nicoleta Nastase, Raluca Amalia Ceausu, Marius Raica, Catalina Poiana

**Affiliations:** 1 Department of Endocrinology, ‘Carol Davila’ University of Medicine and Pharmacy, 020021 Bucharest, Romania; 2 ‘C. I. Parhon’ National Institute of Endocrinology, 011863 Bucharest, Romania; 3 Department of Microscopic Morphology/Histology, ‘Victor Babes’ University of Medicine and Pharmacy, 300041 Timisoara, Romania; 4 Angiogenesis Research Centre, ‘Victor Babes’ University of Medicine and Pharmacy, 300041 Timisoara, Romania

**Keywords:** Aggressive prolactinoma, Breast cancer, PRL-secreting PitNET, Pituitary tumor, PRL, Breast cancer

## Abstract

In several studies, hyperprolactinemia has been associated with increased breast cancer risk. Evidence shows that prolactin (PRL) is linked to mammary tumorigenesis, especially in postmenopausal patients, but the data remain controversial. We present a case of a 67 year-old patient with a resistant PRL-secreting PitNET who subsequently developed breast cancer. The patient was known to have persistent high PRL levels despite multimodal treatment (surgery, radiotherapy, and high doses of cabergoline). The tumor specimens obtained after transsphenoidal intervention were histologically and immunohistochemically examined for the following parameters: anterior pituitary hormones, the ki-67 labeling index, CAM 5.2 expression, ER ∝ expression, and somatostatin receptors, which revealed a densely granulated tumor with intense positivity for PRL and ER ∝ , a ki-67 labeling index of 6% and negative MGMT expression. Years later, the patient was diagnosed with breast carcinoma. Histopathological and immunohistochemical examination of the tumor specimen obtained after radical mastectomy confirmed ductal invasive breast cancer with negative immunostaining for prolactin receptors (PLRr) but positive immunostaining for estrogen (ER) and progesterone receptors (PGR) and a ki-67 labeling index of 8%. PRL is involved in mammary development and differentiation, which leads to lactation, the major driver during pregnancy, by regulating ovarian progesterone production. On the basis of the physiological actions of PRL, a role for this hormone in breast cancer has been suggested. Few cases of different types of breast carcinoma associated with hyperprolactinemia due to a pituitary tumor have been reported in the literature. The association between hyperprolactinemia and the risk of breast carcinoma is not well understood. Immunohistochemistry evaluation of PLRr can be helpful to provide information in these cases.

## Introduction

Prolactin is involved in mammary development and differentiation, which leads to lactation [1]. During pregnancy, PRL-mediated signals expand alveolar cells and coordinate their differentiation at birth. Additionally, PRL is the major driver during pregnancy because it regulates ovarian progesterone production [2]. On the basis of the physiological actions of PRL, a role for this hormone in breast cancer has been suggested [3, 4]. Its involvement in tumorigenesis is linked to prolactin receptors (PRLrs), which can activate the transcription factor STAT5 [5]. Prolactin binds to PRLr and activates JAK kinases via transphosphorylation, leading to STAT5 tyrosine phosphorylation (pSTAT5) [6]. pSTAT5 modulates the expression of key target genes involved in growth, increased differentiation, and survival and has been shown to play a role in resistance to antiestrogen therapy [7, 8].

Almost all evidence shows that high PRL levels are associated with a greater risk of breast cancer in postmenopausal women [9]. The differences between the risk in postmenopausal and premenopausal women are based on the differential actions of prolactin under low and high estrogen, and progesterone and needs a better understanding. The findings are conflicting. There are also important data in the literature indicating that higher levels of plasma PRLare associated with an increased risk of breast cancer in both premenopausal and postmenopausal women, especially for the ER/PGR-positive cancer type [10, 11]. The menopausal status and its relationship with the PRL level remain controversial.

Other data in the literature show that a PRL value greater than 11 ng/ml measured within 10 years of diagnosis was associated with a greater risk of postmenopausal breast cancer in a prospective study [9]. Additionally, a higher PRL level was associated with a more aggressive tumor.

Additionally, other breast cancer risk factors, such as nulliparity and high mammographic breast density, have been shown to be correlated with increased levels of serum PRL [12]. PRL can induce cell proliferation, tumor vascularization, and cell motility, which can promote late-stage carcinogenesis of breast cancer.

Another important factor is the PRLr. The receptor on the surface is a critical determinant of signaling output in response to PRL, and the regulatory elements that control receptor expression are also essential.

## Case report

**Fig. 1:**
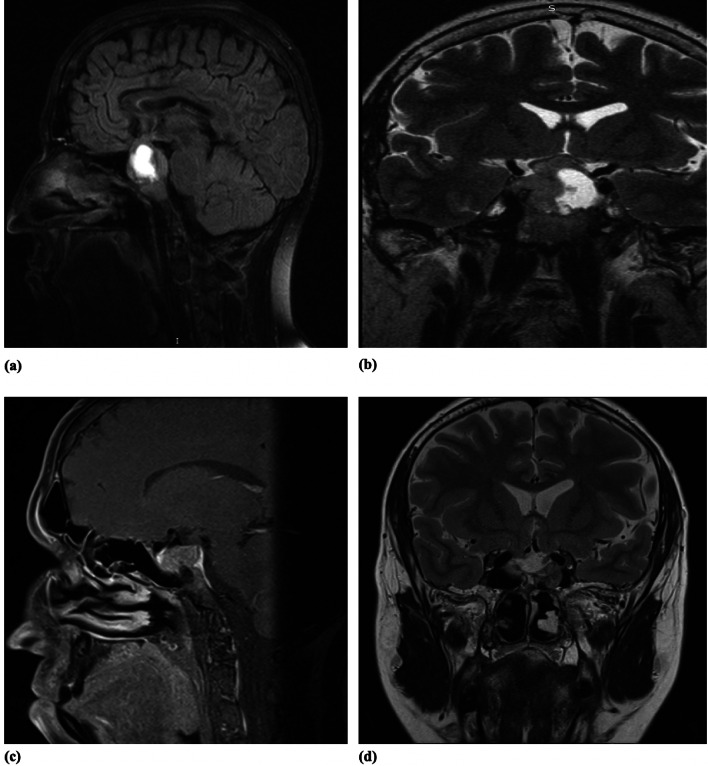
MRI coronal and sagital images. **a** – MRI at the time of diagnosis – hyperintense area at the left side of the pituitary gland of 52/38/52 mm. **b** – Postoperative MRI – no significant regression of the lesion – **c**, **d** – control MRI after radiotherapy and medical treatment

We present the case of a 67 year-old female patient who was diagnosed with hyperprolactinemia in 2016. Magnetic resonance imaging (MRI) revealed a pituitary macroadenoma (52/38/52 mm) with suprasellar extensions, bilateral cavernous sinus invasion and optic chiasm compression (Knosp grade 3A) (Fig. 1). The patient had a history of headache but no nausea, visual field defects, or nipple discharge and no relevant family history. Her PRL level at presentation was 8281 ng/ml (normal range: 2.74–16.64 ng/ml). There were no symptoms of pituitary hormonal deficits. The screening for multiple endocrine neoplasia (MEN) was negative. The patient underwent medical treatment with cabergoline preoperatively for a few months, but owing to the invasive characteristics of the tumor, the medical team decided to recommend surgery. The patient underwent transsphenoidal resection of the tumor in December 2016.

The postoperative tumor blocks were subjected to morphological and immunohistochemical analysis. Antibodies used for IHC: PRL(Dako, Agilent Polyclonal rabbit anti-human with 1:300 dilution and cytoplasmatic expression), GH (Anti-GH, Dako Cytomation, polyclonal rabbit anti-human, dilution 1:400), ACTH (adrenocorticotropic hormone, anti-ACTH, Dako Cytomation, clone C93, dilution 1:50), FSH (folliclestimulating hormone, Anti-FSH, ThermoScientific, clone FSH03, dilution 1:500), LH (luteinizinghormone, Anti-LH, ThermoScientific, clone LH01, dilution 1:500) and TSH (thyroid stimulating hormone, Anti-TSH, ThermoScientific, Mouse Monoclonal Antibody, clone: TSH 01 + TSH 02, diluție1:400), Ki-67 (Thermo Fisher Scientific, MM1, RTU- ready to use—nuclear expression), cytokeratin Cam 5.2 (Diagnostic BioSystems CAM5.2, RTU, cytoplasmatic expression), estrogen receptor α, ER α (6F11, RTU, Leica Biosystems, dilution 1:400), SSTR 2 (recombinant anti-somatostatin receptor 2 antibody – C-terminal abl134152), SSTR 5 (recombinant anti-somatostatin receptor 5 antibody abl09495). IHC analysis was done using Leica Bond Max (Bond Epitope Retrieval Solution 1 și 2). The immunohistochemical expression of PRL, TSH, ACTH, FSH, and LH was analyzed at the cytoplasmatic level, along with the expression of Ki-67 in the nucleus. Stains for the 6 pituitary hormones were scored in a blinded fashion. The proportion score for the anterior pituitary hormones was quantified according to the following criteria: score 0 (0–10% positive cells), score 1 + (10–30% positive cells), score 2 + (30–60% positive cells), and score 3 + (> 60% positive cells). The intensity scores used were from 0 to 3 + (from absent to strongly stained). A staining superior to 10% was considered positive for the purpose of interpreting the results. The nuclear-positive cells for Ki-67 were quantified using optical microscopy (magnification × 20) using Image J version 2.0 (a semiautomatic evaluation, which excluded endothelial and stromal cells’ nuclei).

**Fig. 2:**
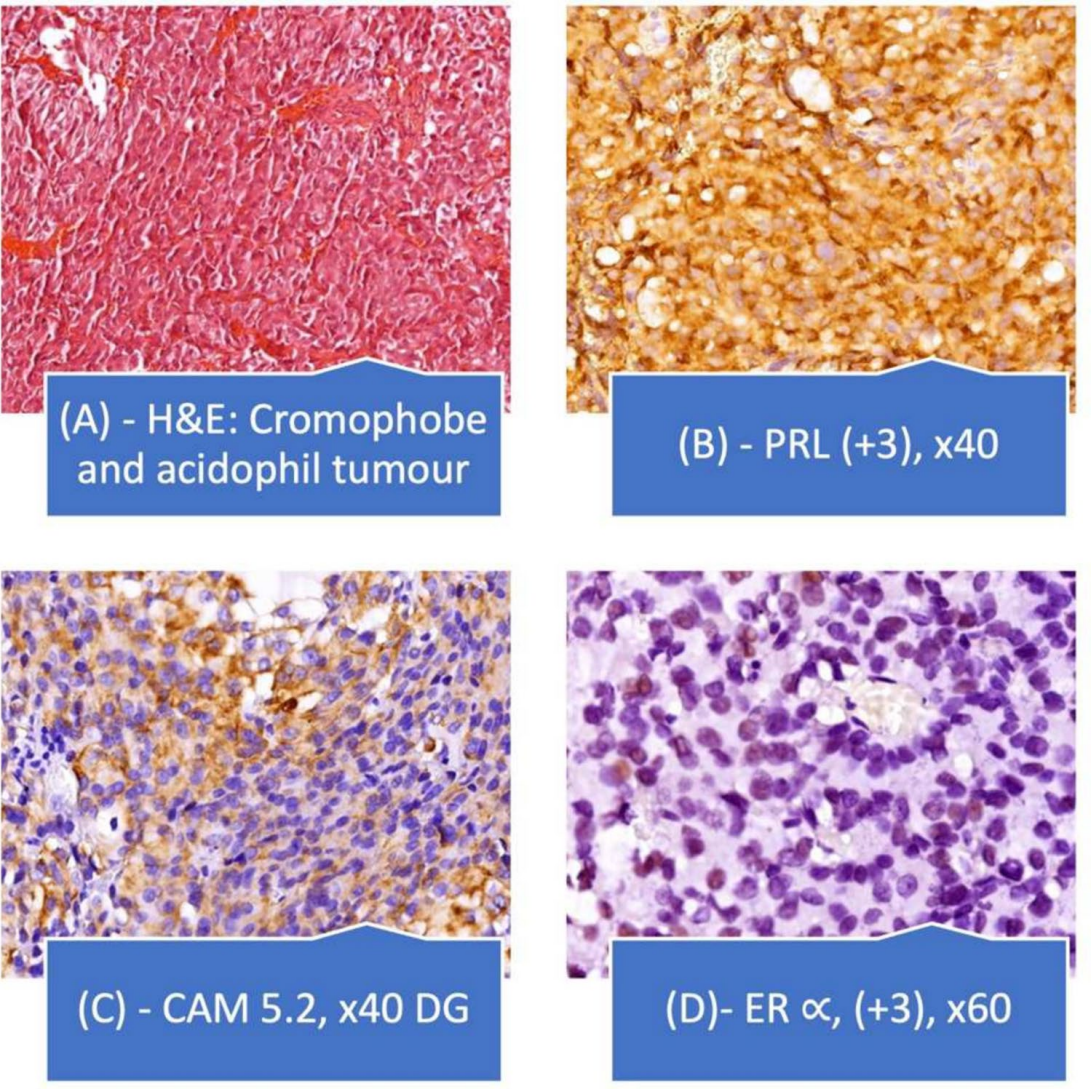
Histological and immunohistochemical examination of the pituitary tumor specimen. PRL-secreting PitNET. **a** Hematoxylin and eosin (H&E) staining (× 20 magnification, chromophobe and acidophil adenoma), **b** positive intense IHC staining for PRL (+ 3, × 40 magnification), **c** CAM 5.2 densely granulated pattern (× 40 magnification), **d** ER ∝ intensely positive (+ 3, × 60 magnification)

Histological analysis revealed a solid mixed, acidophilic and chromophobe adenoma that was densely granulated (based on CAM 5.2 expression) with positive intense immunostaining for PRL, a ki-67 labeling index of 6% and positive ER α expression. Additionally, the immunohistochemical analysis revealed positive staining for somatostatin receptor type 5 (+ 1) and a negative reaction for somatostatin receptor type 2 (Fig. 2). Postoperative MRI revealed no significant regression of the lesion. The postoperative hormonal work-up revealed persistent high PRL levels, and the patient was given a gradually increasing dose of cabergoline up to a maximum of 6 mg/week. The prolactin levels under treatment remained high, and there was no significant reduction in the number of pituitary lesions. Owing to a lack of efficacy, medical treatment was stopped, and the patient was referred for radiotherapy (3D-CRT (three-dimensional conformal radiation therapy) with a total dose of 50.4 Gy in 28 fractions. Postradiotherapy, there was a slight reduction in the PRL, and cabergoline treatment was reinitiated. Other treatments, such as somatostatin analogs or temozolomide, have not yet been considered (the patient was evaluated periodically while waiting for the effect of radiotherapy).

**Fig. 3:**
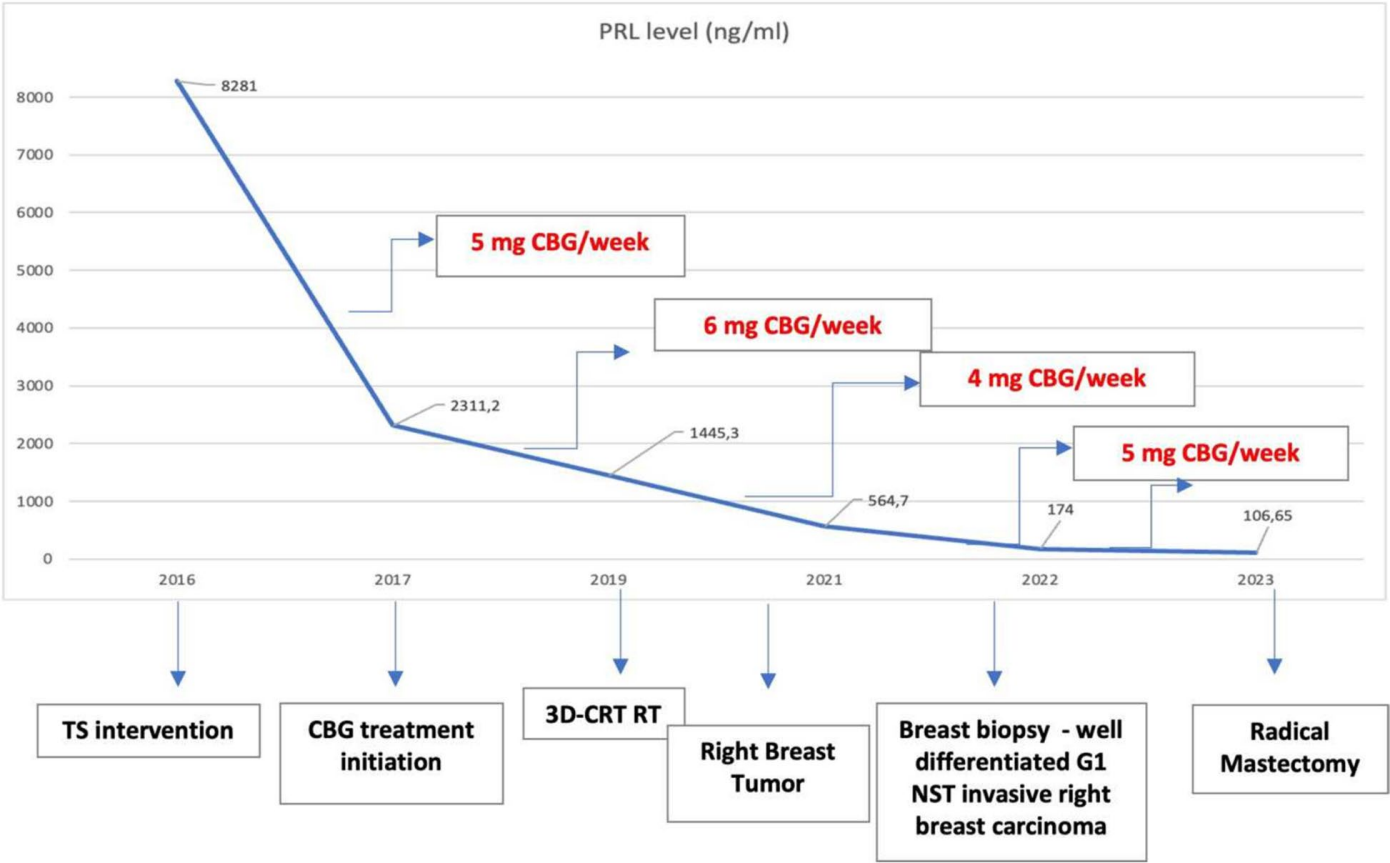
Postoperative prolactin levels during the treatment with cabergoline. *PRL* prolactin, *TS* transsphenoidal, *CBG* cabergoline, *3D-CRT RT* three-dimensional conformal radiation therapy, *NST* no special type; * the first four PRL values are after dilution

**Fig. 4:**
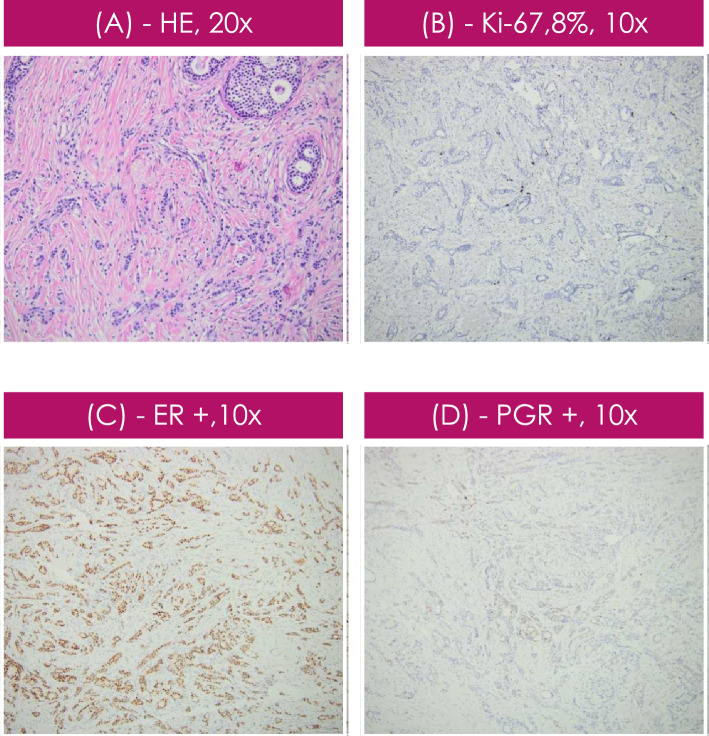
Histological and immunohistochemical examination of the breast tumor specimen. Ductal invasive Breast Carcinoma NOS (no otherwise specified): **a** Hematoxylin and eosin (H&E) staining: solid and cribriform pattern. (× 20 magnification), **b** ki-67 labelling index of 8% (× 10 magnification), **c** positive estrogen receptor (× 10 magnification), **d** positive progesterone receptor (× 10 magnification). Also, Her2 negative and p63 positive in myoepithelial cells

Control MRI revealed no significant regression of the residual tumor mass. After 5 years of cabergoline treatment, the PRL level decreased to 106.65 ng/ml (normal values: 2.76–19.64) under 5 mg of cabergoline per week, and MRI revealed necrosis of the residual tumor mass (11/10/11 mm) with retrosellar extension (Figs. 3, 4).

The patient had a history of left breast surgery for 2 benign tumors confirmed by histopathological examination. At the initial presentation, a breast mammography was performed, which revealed abnormal density in the left retroareolar region. A breast MRI was performed, which did not reveal any alterations. She had periodic breast imaging evaluations. In 2019, breast mammography revealed a right breast with abnormal density and bilateral axillary lymph nodes and microcalcifications (BI-RADS ACR 3.3). The patient underwent additional investigations: a breast MRI revealed several masses in the right breast of 18/23/18.4 mm in the superior external quadrant at 11:00 and another mass at 8:00 in the infero external quadrant of 6.5/5.1/6.1 mm. The patient underwent a systemic examination to rule out distant metastases and other primary tumors. A breast biopsy revealed a well-differentiated G1 NST (no special type) invasive right breast carcinoma. Immunohistochemical evaluation revealed positive estrogen and progesterone receptor (ER, PGR) expression and with a ki-67 labeling index of 8%. HER2 expression or PRLr was not evaluated at this time. Treatment with letrozole was initiated, the patient was under oncological observation and modified radical mastectomy was recommended. The pathology specimen obtained after mastectomy revealed infiltrating right ductal breast carcinoma with positive estrogen and progesterone receptors, a ki-67 labeling index of 8%, and negativity for prolactin receptors and HER2 (0).

Presently, the patient has a lower PRL of 106.65 ng/ml (normal values: 2.76–19.64) under 5 mg of cabergoline per week, and the last pituitary MRI showed a smaller pituitary mass (Table 1).

**Table 1:** Cases reported in the literature, including male and female patients diagnosed with PRL-secreting PitNETs and breast carcinoma

Case report, year	Age	Sex	Breast carcinoma histology features	Breast localisation	IHC features beast specimen	HER2 status	Breast carcinoma treatment	Timing of breast and pituitary tumor diagnosis
Daniels et al., 1976	33	Female	IDC	Left	–	N/A	Mastectomy + RT	Pituitary Tumor, Breast Carcinoma (6 years)
Buytaert et al., 1981	29	Female	IDC	Right	–	N/A	Mastectomy + CHT	Pituitary Tumor, Breast Carcinoma (2 years)
Olsson et al., 1984	48	Male	NIDC	Bilateral	–	N/A	Mastectomy + RT	Pituitary Tumor, Breast Carcinoma (26 years, second breast tumor at 36 years)
Theodorakis et al., 1985	31	Female	IDC	Left	ER ( +), PGR ( +)	N/A	Mastectomy	Pituitary Tumor, Breast Carcinoma (3 years)
Haga et al., 1993	68	Male	IDC	Left	ER (+ +), PGR ( +)	N/A	Mastectomy + CHT + HT	Synchronous
Strungs et al., 1997	52	Female	IDC	Right	ER ( +)	N/A	Mastectomy + HT	Synchronous
	34	Female		Right	ER ( +)	N/A	Mastectomy + RT	Pituitary Tumor, Breast Carcinoma (9 years)
Volm et al., 1997	70	Male	IDC	Bilateral	ER ( +), PGR ( +)	N/A	Mastectomy + HT	Pituitary Tumor, Breast Carcinoma (7 years)
Forloni et al., 2001	45	Male	L-IDC R-DCIS	Bilateral	ER ( +), PGR ( +)	N/A	Mastectomy	Synchronous
Sato et al., 2006	43	Female	IDC	Left	ER ( +), PGR ( +)	HER2 (-)	Mastectomy, CHT + HT	Pituitary Tumor, Breast Carcinoma (16 years)
Mallawaarachch et al., 2011	56	Male	DCIS	Right	–	N/A	Microdochectomy	Pituitary Tumor, Breast Carcinoma (3 years)
Poiana C., Musat M, 2013	40	Female	Mucinous Breast Cancer with neuroendocrine differentiation	Unilateral	PLRr ( +)	N/A	Mastectomy	Pituitary Tumor, Breast Carcinoma
Zheng Y. et al., 2017	28	Female	IDC	Left	ER ( +), PGR ( +)	HER2 (-)	Mastectomy	Synchronous
Abghari et al., 2018	52	Male	DCIS	Left	ER ( +), PGR ( +)	N/A	Mastectomy	Pituitary Tumor, breast Carcinoma (10 years)
Hao et al., 2020	51	Male	IDC	Right	ER (+ + +), PGR (+ + +)	HER2 (-)	Mastectomy + CHT	Synchronous

*IDC* invasive ductal carcinoma, *NIDC* noninvasive ductal carcinoma, *DCIS* ductal carcinoma in-situ, *ER* estrogen receptor, *PGR* progesterone receptor, *PLRr* prolactin receptor, *HER2* human epidermal growth factor receptor 2, *CHT* chemotherapy, *HT* hormonal therapy, *RT* radiotherapy, *IHC* immunohistochemistry, *N/A* unknown

## Discussion

Several previous studies have shown that hyperestrogenism induced by endocrine disorders may be a major risk factor for breast cancer. However, obesity, family history, radiation exposure, and other factors are associated with breast cancer. To date, all the evidence available on breast cancer and PRL-secreting PitNETs has been from retrospective analyses or case reports.

The influence of PRL on breast cancer remains a subject of debate: some studies have shown that prolactin levels are not significantly higher in patients with breast cancer than in control individuals. One study reported a standardized mortality ratio of 1.07 (95% confidence interval: 0.5–2.03) among 1.342 patients with breast cancer who received treatment for hyperprolactinemia [12]. Another study involving 1,400 patients revealed that prolactin was associated with the occurrence of breast cancer, especially in postmenopausal women (relative risk: 1.37, *p* < 0.05) and in patients with ER + status (relative risk: 1.28, 95% confidence interval: 1.07–1.54, *p* = 0.003) [9].

PRL and estrogens play very important roles in normal mammary gland growth and development. The theory of PRL as a causative factor in breast cancer was initially suggested on the basis of studies that included mouse models. High circulating levels of PRL increase the synthesis and expression of PLRr in malignant mammary tissue, increasing DNA synthesis in breast cancer cells [13].

Other studies have evaluated the associations between PRL levels and several well-confirmed breast cancer risk factors, such as parity and age at menarche. Overall, lower exposure to estrogens and androgens in premenopausal women is hypothesized to decrease breast cancer risk. A strong association was found between PRL-secreting PitNETs and a family history of breast cancer [14, 15].

Cases of breast cancer were reported both in women and men. We identified 15 cases of breast cancer in patients with prolactinoma, 7 in men and 8 in women. In the majority of cases, we observed a latency period of diagnosis of several years, up to 36 years, and in almost all cases, the pituitary tumor was first diagnosed. Histopathological analysis revealed that the majority of patients were diagnosed with invasive ductal carcinoma with positive immunostaining for estrogen and progesterone receptors. One case with neuroendocrine differentiation of a unilateral breast tumor was reported in a 40 year-old female with positive immunostaining for PRLr.

Benign breast disease is a major risk factor that doubles the risk of subsequent breast cancer. Data show that the increase in risk is sustained and for 20 years after diagnosis and also, women who had a proliferative benign disease have a higher long-term risk than those with non-proliferative disease. Other studies show a 70% higher risk of breast cancer in patients with prior breast disease, than those without [16] PLRrs are a major mediator of the cellular effects of PRL. The Jak-STAT, Ras-MAPK, and PI3K-Akt pathways are the major mechanisms that mediate the effects of PRL [17]. PRL activates the Ras-Raf-MAPK pathway in mammary tumor cell lines, which signals cell proliferation [18]. Additionally, PRL activates other MAPKs, such as JNK, which impact proliferation and apoptosis [19–21]. Other kinases, such as c-Src, are activated in response to PRL and interact with PRLr-mediated signaling [22].

There are various isoforms of PRLr in various species. The expression of PRLr has been studied in normal, benign, and malignant breast tissue, and one immunohistochemical study that evaluated paraffin wax-embedded sections of 102 breast biopsies revealed that the receptor was expressed in more than two-thirds of female breast carcinomas and that positivity was correlated with moderate and strong staining for the ER in tissue sections [23]. Additionally, positive expression of PRLr was correlated with prognosis. In one study that evaluated the immunohistochemical analysis of PRLr protein expression levels via a tissue microarray of 102 cases, PRLr expression was found to be significantly downregulated in invasive breast cancer and was associated with lymph node negativity and low-grade well-differentiated tumors [24]. PRLr was found to be an independent predictor of favorable prognosis in human breast cancer patients.

Multiple promoters control human PRLr expression at the transcriptional level. Each promoter directs transcription/expression of a specific non-coding exon 1, a common non-coding exon 2, and coding exons E3-11. The identification of exon 11 of PRLR led to the discovery of alternative spliced products and two novel short forms (SF) that can inhibit the long form (LF) of PRLr activity, which is relevant in physiological regulation and breast cancer.

Constitutive LF and SF homodimers and heterodimers. Both forms, as dimers, are capable of ligand binding and PRL-induced phosphorylation of JAK2, but only LF can activate downstream STAT5 signaling.

Male breast cancer is a rare form of breast cancer, data from the literature show that the risk rises with age [25]. Prospective studies and clinical trials on breast cancer treatments have often excluded male participants. It seems that 15%–20% of patients have a family history of breast or ovarian cancer, and approximately 10% of the patients have a genetic predisposition. BRCA2 and Klinefelter’s syndrome were associated with an increased risk of male breast cancer [26]. We searched the literature and we found 7 cases reported in male patients diagnosed with prolactinoma. Hyperprolactinemia occurred before the diagnosis or concurrent with the diagnosis of breast cancer in all cases.

The role of estrogen in breast cancer has been deeply studied, but the role of testosterone has not been established. One proposed theory is that excess circulating testosterone can be aromatized into estrogen, which has been shown to increase human prolactin receptor gene expression within the peripheral tissues, which in turn directly stimulates breast tumor cell proliferation [27, 28].

Transgenic male mice that were treated with the mammary-selective, estrogen-insensitive promoter neu-related lipocalin (NRL), which drives PRL expression, and did not develop mammary tumors. However, in cooperation with transforming growth factor-α (TGF-α), PRL mammary tumors were induced in 100% of male bitransgenic mice. Similar to disease in human males, these tumors expressed variable levels of ER α and androgen receptors. Male breast tumors demonstrate high levels of ER α expression, similar to those in postmenopausal women. Like female patients, male patients exhibit resistance to antiestrogens, such as tamoxifen, or develop resistance after treatment. In vivo, PRL in combination with TGF-α induces ER α-positive, but estrogen-insensitive, disease [29–31].

Another association was observed between invasive breast cancer risk in postmenopausal women with high circulating PRL, particularly for ER-positive disease. Also, PRL/PRLR is expressed in 95% of mammary tumors and 60% of male breast carcinomas [32]. PRL has an essential role in the upregulation of the PRLr promoter, which involves the requisite participation of E2/ER α at the PRL promoter along with STAT5a [33].

**Fig. 5:**
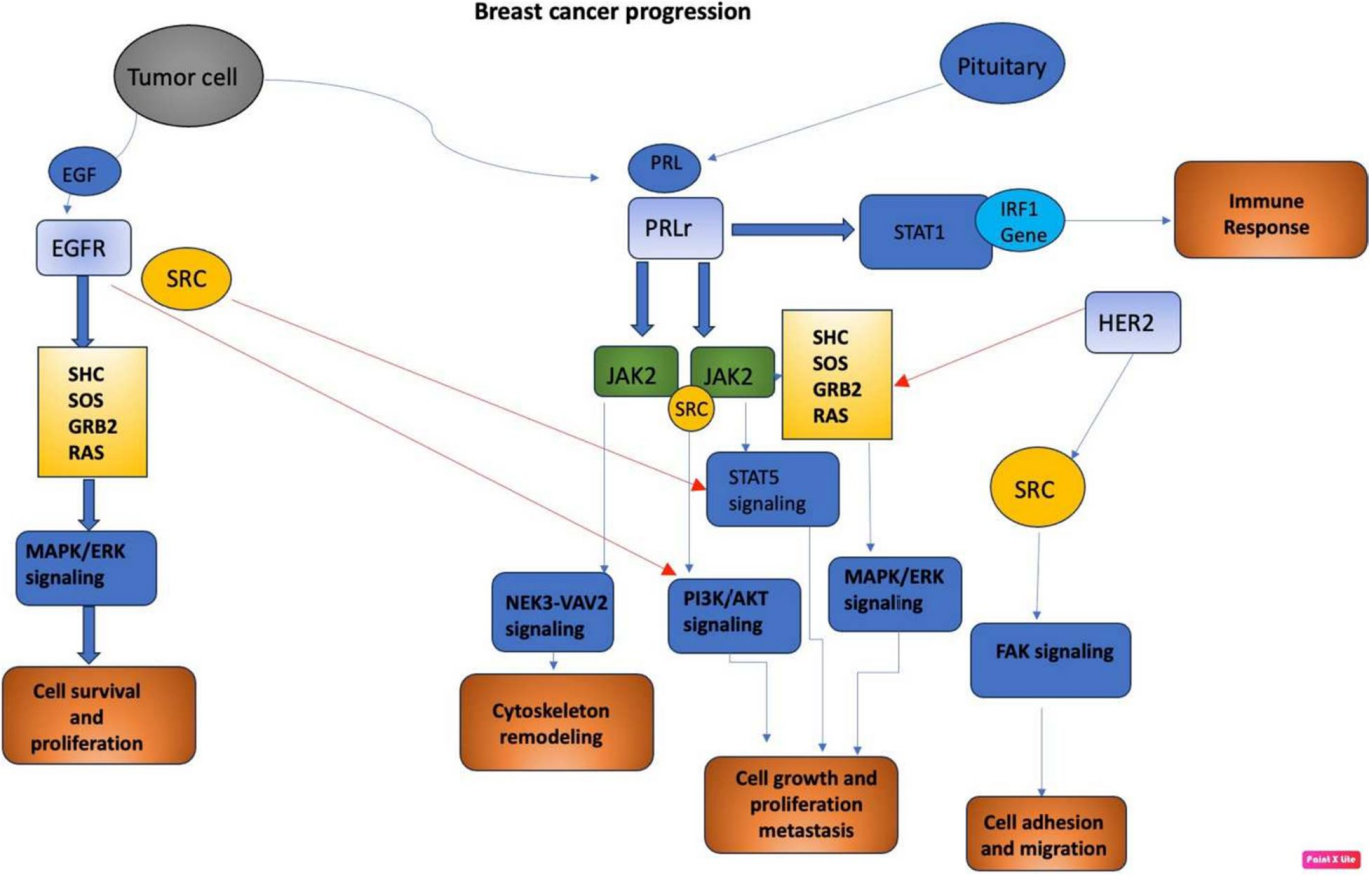
PRLr, EGFR and HER2 signaling in breast cancer (adapted from Kavarthapu R, Anbazhagan R, Dufau ML. Crosstalk between PRLR and EGFR/HER2 signaling pathways in breast cancer. Cancers (Basel) (2021) 13(18):4685). EGF is released by stromal microenvironment surrounding the breast tumor and activates signaling cascades that overlap with PRLr signaling cascades. PRLr is activated by the PRL secreted by breast tumor cells. PRL stimulates HER2 and EGFR signaling pathways via JAK2. EGF EGFR activates STAT5 signaling indirectly via s-SRC. Adapter proteins: SOS, SHC, GRB2. Legend: *EGF* epidermal growth factor, *EGFR* epidermal growth factor receptor, *PLRr* prolactin receptor, *SRC* Src protein-tyrosine kinase, *SHC* SHC-transforming protein, *SOS* son-of-sevenless protein, *GRB2* growth factor receptor bound protein 2, *MAPK/ERK* classical mitogen-activated protein kinase pathway, *FAK* focal adhesion kinase

Other receptors that play a key role in breast cancer tumorigenesis are EGFR (epidermal growth factor receptor) and HER2. EGF released by the stromal microenvironment surrounding the breast tumor activates signaling cascades that overlap with PRLr signaling cascades upon activation with PRL secreted by breast tumor cells. PRL stimulates HER2 and EGFR signaling pathways via JAK2. EGF/EGFR also activates STAT5 signaling indirectly via s-SRC. This crosstalk between receptors can increase progression of breast tumor and endocrine resistance (Fig. 5) [34].

Another event that contributes to the progression and motility of human breast cancer is the activation of the VAV family of guanine nucleotide exchange factors. The PRL-mediated activation of Nek3 contributes differentially to VAV2 signaling pathways involving Rac1 and signal transducer and activator of transcription 5 and implicates Nek3 during PRL-mediated actions in breast cancer [35].

In conclusion, more studies are needed to understand the mechanisms that regulate PRLr expression and function, but the causal role of the PRLr signaling axis in the pathogenesis of breast carcinoma is well established. A possible clinical application should encourage research in this area. On the basis of the observation that, in several immunohistochemical studies of patients with breast carcinoma, PRLr is expressed at high levels, the evaluation of PRLr expression should be considered in these patients and should be mandatory in patients diagnosed with PRL-secreting PitNETs. The impact of PRLr on prognosis is still under debate, but some studies have shown that PRLr can be a predictor of a favorable prognosis. Our patient experienced favorable evolution after breast cancer treatment concurrently with normalization of prolactin levels under cabergoline treatment. This case is intriguing and provides new evidence that resistant PRL-secreting PitNETs can be associated with the development of breast cancer. The patient had a history of breast masses that have undergone changes over the years and developed into tumor masses. The patient history of breast surgery before the prolactinoma diagnosis, and the high persistent PRL values despite the treatment are factors that have increased the risk of breast carcinoma development.

## Data Availability

Derived data supporting the findings of this study are available from the corresponding author, RID-S, upon request.
